# Comprehensive Analysis of Breastfeeding's Influence on Child Health Outcomes: A Cross-Sectional Study

**DOI:** 10.7759/cureus.64194

**Published:** 2024-07-09

**Authors:** Sidra Kamal, Ruchira Clementina, Mubashir Ali Aftab, Aqsa Haider, Muhammad Ibrahim, Nimra Abid, Ayaz Ali, Ashraf Ali, Nida Gul, Afaq Ahmad

**Affiliations:** 1 Internal Medicine, Karachi Medical and Dental College, Karachi, PAK; 2 Medicine, Government Medical College, Nizamabad, IND; 3 Internal Medicine, Fazaia Medical College, Islamabad, PAK; 4 Internal Medicine, Khyber Medical College, Peshawar, PAK; 5 Internal Medicine, Rehman Medical Institute, Peshawar, PAK; 6 Surgery, Institute of Medical Sciences, Khyber Medical University, Kohat, PAK; 7 Medicine, Khyber Medical College, Peshawar, PAK

**Keywords:** cross secional study pakistan, developmental delays, cognitive development, infectious diseases, formula feeding, partial breastfeeding, exclusive breastfeeding, developmental outcomes, child health, breastfeeding practices

## Abstract

Background

Breastfeeding is recognized as a crucial determinant of child health and development, yet its multifaceted effects remain underexplored in many contexts. This cross-sectional study investigates the association between breastfeeding practices and various health and developmental outcomes in infants and young children, focusing on exclusive breastfeeding, partial breastfeeding, and formula feeding. Done at Khyber Teaching Hospital, Pakistan, the research aims to provide comprehensive insights into the nuanced impacts of breastfeeding on child well-being.

Objectives

This study aims to assess the association between breastfeeding duration and practices with the incidence of infectious diseases in infants and young children. It investigates the relationship between different breastfeeding practices: exclusive breastfeeding, partial breastfeeding, and formula feeding and cognitive development outcomes in early childhood. Additionally, the study evaluates the role of breastfeeding in the development of motor skills in infants and young children.

Methodology

A cohort of 390 participants, aged one month to three years, participated in the study. Data collection encompassed parental interviews, clinical assessments using standardized tools such as the Bayley Scales of Infant Development, and reviews of medical records. Statistical analyses, including frequency analysis and chi-square tests, were conducted to elucidate the relationships between breastfeeding practices and health outcomes.

Results

Exclusive breastfeeding exhibited a significantly lower incidence of infectious diseases compared to partial breastfeeding and formula feeding. Specifically, among exclusively breastfed children, incidences of colds, pneumonia, and diarrhea were 32%, 39.7%, and 40%, respectively. These rates were notably higher in partially breastfed and formula-fed children. Cognitive development outcomes also varied significantly across feeding groups. Exclusively breastfed children demonstrated superior cognitive performance, with 34.2% rated above average, compared to only 6.5% in the formula-fed group. Additionally, the prevalence of developmental delays was lowest among exclusively breastfed children (14.1%), contrasting with 62.8% in the partial breastfeeding group and 77.0% in the formula-feeding group.

Conclusions

The study underscores the pivotal role of exclusive breastfeeding in promoting optimal child health and development. Exclusive breastfeeding is associated with significantly reduced incidences of infectious diseases, superior cognitive development outcomes, and a lower prevalence of developmental delays. These findings highlight the importance of supportive interventions and policies aimed at encouraging exclusive breastfeeding practices, ultimately enhancing child well-being and developmental trajectories.

## Introduction

The nutritional status of children under two years old, as well as their risk for infectious diseases and mortality, is significantly influenced by the feeding practices used during infancy and early childhood [1]. The World Health Organization (WHO) advises that breastfeeding should start as soon as the baby is born and continue for at least two years. It should be done exclusively for the first six months of the child's life before safe, nutrient-rich complementary foods are introduced [2].

Infants who are breastfed have better cognitive outcomes and reduced incidence of infectious disorders and childhood obesity compared to non-breastfed babies [3]. Moreover, breastfeeding protects mothers against type 2 diabetes, cardiovascular diseases, and cancers of the breast and ovary [4].

According to US breastfeeding data for babies born in 2017, 85% of mothers started breastfeeding at the age of two days, but 20% of them gave formula before that time, and less than half of them were exclusively breastfeeding by the time their babies were three months old [5]. Even though human milk is proven to have health benefits for babies, only 25% of babies are exclusively breastfed by the time they are six months old, and 60% of mothers fall short of their nursing targets [6]. Inadequate breastfeeding rates in the United States are projected to result in an annual increase in medical expenses for mothers and newborns of over $3 billion [7].

Numerous studies have looked at the causes of suboptimal breastfeeding outcomes (non-exclusive breastfeeding [EBF] and/or breastfeeding cessation). These causes include obstacles such as unsupportive work and hospital policies, challenges with lactation, and concerns about the growth and nutrition of the infant from the mother or healthcare provider [8].

Compared to women in Australia, fewer women in Ireland and the UK breastfeed. Context is crucial because it shows that breastfeeding interventions must be customized for specific contexts and take into account local cultural and societal norms that may influence behavior. To account for the interplay between systems, these interventions must also be responsive. This research expands on earlier findings and provides compelling evidence that breastfeeding-related factors are multifaceted, complex, and country-specific. This implies that any effective interventions and strategies to increase breastfeeding rates must be adapted to local needs and take a comprehensive approach to the various systems that surround the mother and child [9].

Despite being a popular practice in India, breastfeeding is linked to misconceptions and superstitions, such as the idea that colostrum is harmful to the infant and that breast milk is insufficient during the first three days after delivery [10]. The Government of India encourages commencing breastfeeding within one hour of labor. However, just 22% of infants received breastfeeding within one hour, while over one-third (35.8%) received it after 24 hours following delivery [11].

A study in India found that although breastfeeding was widespread in the research location, only 22% of mothers started within 1-3 hours. Approximately 45% of newborns received pre-lacteals, including honey (25%), whereas 85% received colostrum. One of the reasons for mothers not giving colostrum was the elders' advice [12].

A study in the UAE, the first cohort study to look at multilevel factors of breastfeeding behaviors in the UAE, found several major modifiable factors for suboptimal breastfeeding, including breastfeeding self-efficacy (BSE), maternal postpartum depression, employment, and social support from family and friends. The findings suggest that UAE health authorities' efforts to encourage EBF should extend beyond hospital settings and involve multimodal measures to preserve EBF for the first six months after birth. Early detection of potential depressive symptoms with the Edinburgh Postnatal Depression Scale (EPDS), as well as perinatal evaluation of BSE with the Breastfeeding Self-Efficacy Scale-Short Form (BSES-SF) and subsequent referral for official antenatal or postnatal risk assessment, should help lead intervention programs to improve EBF rates in the UAE. Furthermore, community-based groups must involve family members, including husbands, grandmothers, sisters, and friends [13].

A study conducted in Pakistan concluded that the prevalence of EBF was 53.6%. EBF lowers the risk of childhood diseases. Possible explanations for the observed link include the contents of human milk, which provide nutrients required to meet an infant's demands throughout the first few months of life. In addition, continuous breastfeeding lowers exposure to other contaminated foods, ensuring appropriate nutrition for the infant [14].

Infants transitioning from EBF to follow-on formula should be monitored closely for infectious illness incidence. Breastfeeding has been found to have a variety of benefits for newborns, including protection against infectious and allergic illnesses, for example, studies have shown that breastfed infants have lower rates of respiratory infections like bronchiolitis and pneumonia, fewer instances of gastroenteritis, and a reduced risk of developing atopic dermatitis and asthma compared to formula-fed infants. These effects are probably mediated in part by modification of the gut microbiota [15].

The impact of feeding type on infant health and development was first noted over seventy years ago, with evidence indicating that breastfed infants exhibit superior cognitive function compared to those who are not breastfed [16]. The long-term positive effects of breastfeeding on a child’s mental health can be attributed to the richness of maternal milk, which is abundant in fatty acids and other bioactive components. These essential nutrients are vital for the brain development of infants, supporting their cognitive growth and potentially leading to better mental health outcomes as they mature. By providing these crucial building blocks for brain development, breastfeeding may offer lasting benefits that extend well beyond infancy, contributing to improved mental health throughout the child's life [17].

Docosahexaenoic acid (DHA) and arachidonic acid (ARA) are two fatty acids that are linked to nerve cell growth, retinal function, and brain function. Breast milk contains both, whereas infant formula and cow's milk do not. Both have been demonstrated in trials to improve vision and some motor reflexes in newborns and young toddlers [18].

Breastfeeding is widely recognized for its health benefits, yet its specific impacts on various aspects of child development require further exploration. Understanding the relationship between breastfeeding duration and practices with the incidence of infectious diseases can provide critical insights into optimal infant nutrition strategies to enhance immune protection. Additionally, investigating how different breastfeeding practices, such as EBF, partial breastfeeding, and formula feeding, affect cognitive development can help delineate the cognitive advantages associated with breastfeeding. Finally, evaluating the role of breastfeeding in motor skill development in infants and young children can inform recommendations for early childhood care that promote holistic growth and development. This study aims to provide comprehensive evidence on these associations to support informed healthcare decisions and parental guidance.

This research aims to explore the impact of breastfeeding practices on various health and developmental outcomes in infants and young children. Specifically, the study focuses on the following objectives:

(1) Assessing the association between breastfeeding duration/practices (exclusive, partial, or formula feeding) and the incidence of infectious diseases (diarrhea, colds, pneumonia, and ear infections)

(2) Exploring the potential impact of breastfeeding practices on cognitive development outcomes in early childhood, examining the relationship between the type of breastfeeding and cognitive performance indicators

(3) Evaluating the role of breastfeeding in the development of motor skills and determining whether different breastfeeding practices are associated with developmental delays in motor skills

## Materials and methods

Study setting

This study was conducted at Khyber Teaching Hospital, Peshawar, Khyber Pakhtunkhwa Province, Pakistan. Khyber Teaching Hospital was chosen due to its diverse patient population and comprehensive healthcare facilities, which provide a representative sample for the study.

Study type

This cross-sectional study was designed to assess the association between breastfeeding duration/practices and child health outcomes at a single point in time. Data were collected at a specific time point, capturing information on breastfeeding practices and child health outcomes simultaneously.

Participants

The inclusion criteria for this study were children aged between one month and three years, whose mothers were willing to participate, and children without any congenital defects affecting health outcomes. Exclusion criteria included children aged below one month or above three years, those with developmental disorders affecting health outcomes, and children whose mothers were unwilling to participate or unable to provide informed consent.

Sample size

To determine the sample size, we utilized the formula SS = *Z*^2⋅*P* (1 − *P*)/*D*^2, where SS is the sample size, *Z* is 1.96 (reflecting the 95% confidence level), *P* is the expected prevalence or proportion (approximately 53% from previous studies), and *D* is the margin of error (0.05). By inputting these values into the formula, we calculated an approximate sample size of 384 participants.

Sampling technique

We used convenience sampling, where participants were selected based on their availability and accessibility to the researchers. This method involved recruiting individuals who were easily accessible and willing to participate in the study, such as attendees at pediatric clinics, community health centers, or childcare facilities within the study area.

Variables

The independent variables in this study included breastfeeding duration and exclusivity, as well as the type of breastfeeding practices (EBF, partial breastfeeding, and formula feeding). Dependent variables included the incidence of infectious diseases, cognitive development outcomes, and developmental delay. Control variables encompassed maternal factors (age, education, and socioeconomic status), child factors (birth weight and gestational age), and environmental factors (exposure to pollutants and access to healthcare).

Data collection methods

Information on breastfeeding practices (duration and exclusivity), feeding practices, and child health outcomes was collected through parental interviews and questionnaires (for the survey questionnaire, see Appendix). Cognitive development was evaluated using standardized tools such as the Bayley Scales of Infant Development, and developmental delays were identified using the developmental milestone table and the motor scale of the Bayley Scales. Additional relevant data were gathered through a review of medical records. By implementing these methods, we aimed to provide a comprehensive understanding of the impact of breastfeeding practices on the health and development of young children in this region.

Data analysis

The statistical analyses were performed using SPSS software, version 20 (IBM Corp., Armonk, NY). For this study, a *P*-value of less than 0.05 was considered to indicate statistical significance. The data analysis was conducted using several statistical methods to explore the relationship between breastfeeding practices and various health and developmental outcomes in infants and young children. Initially, frequency analysis was performed to determine the distribution of breastfeeding practices (EBF, partial breastfeeding, and formula feeding) among the study population. Bar charts were created to visually represent these distributions and the incidence of infectious diseases, cognitive development outcomes, and motor skills development across different breastfeeding groups. To assess the statistical significance of the observed relationships, chi-square tests were applied. These tests helped determine whether there were significant associations between the type of breastfeeding practice and the incidence of infectious diseases, cognitive development outcomes, and motor skills development. The results of these analyses provide insight into the potential impact of breastfeeding practices on child health and development.

## Results

Demographic characteristics of participants and breastfeeding duration

**Figure 1:**
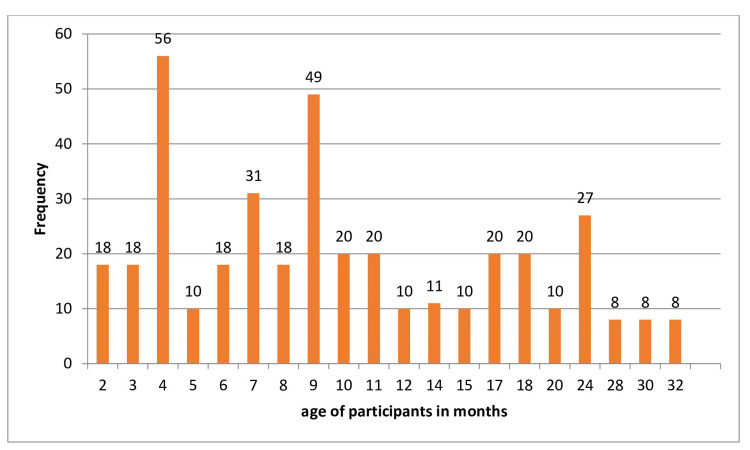
Age-frequency graph.

The study included a total of 390 participants. The age of the participants ranged from two to 32 months, with a mean age of 11.25 months (standard deviation [SD] = 7.703). This indicates a broad age distribution among the participants, encompassing both infants and toddlers. In terms of breastfeeding duration, the participants exhibited a wide range of practices. The duration of breastfeeding varied from 0 to 24 months, with a mean duration of 6.39 months (SD = 6.009). This range reflects the diversity in breastfeeding practices within the study population, from those who were not breastfed at all to those who were breastfed for up to two years (Table 1; Figure 1).

**Table 1:** Demographic characteristics of participants and breastfeeding duration. *N*, number of participants; SD, standard deviation

	N	Range	Maximum	Minimum	Mean	SD
Age of participants in months	390	30	32	2	11.25	7.703
Breastfeeding duration in months	390	24	0	24	6.39	6.009

Distribution of breastfeeding practices

**Figure 2:**
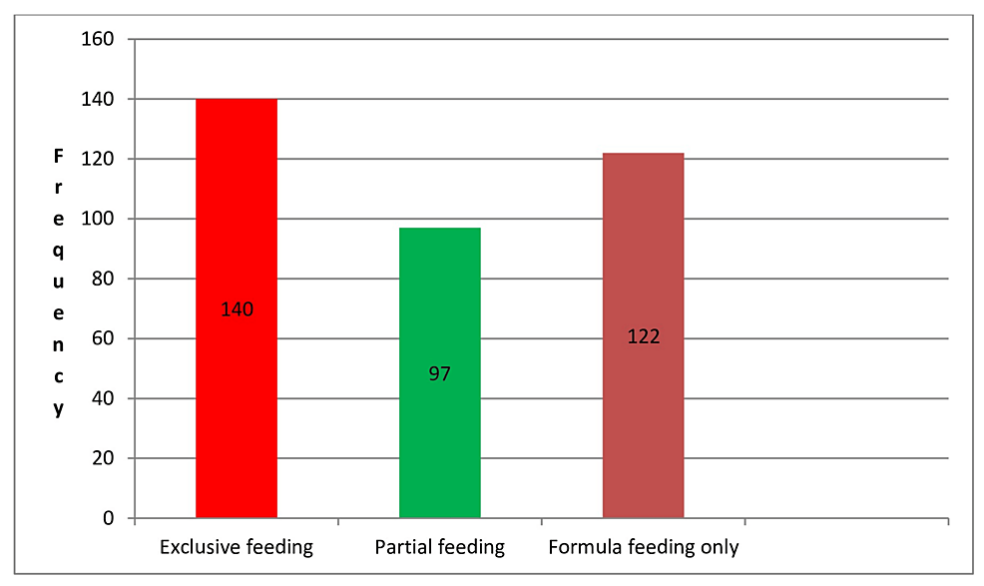
Distribution of breastfeeding practices.

Table 2 presents the distribution of breastfeeding practices among the participants. The breastfeeding practices were categorized into three groups: EBF, breastfeeding supplemented with formula, and formula feeding only. Out of the 390 participants, 140 (35.9%) were exclusively breastfed. This means these infants received only breast milk without any supplemental feeding. A total of 97 participants (24.9%) were partially breastfed, indicating that these infants received a combination of breast milk and other types of feeding such as formula or solid foods. The remaining 122 participants (31.3%) were fed exclusively with formula, without any breast milk. This distribution highlights the varying breastfeeding practices among the participants, with a significant portion being either exclusively breastfed or exclusively formula-fed, and a smaller proportion receiving a combination of both (Table 2; Figure 2).

**Table 2:** Distribution of breastfeeding practices.

Breastfeeding practices	Frequency (*n*)	Percentage (%)
Exclusive breastfeeding	140	35.9%
Partial breastfeeding	97	24.9%
Formula feeding only	122	31.3%

Frequency of infectious diseases

Table 3 summarizes the prevalence of various infectious diseases among the participants. The data indicated that 47.4% of the participants experienced colds, 53.6% had pneumonia, 60.8% suffered from diarrhea, and 12.1% had ear infections. Additionally, other health issues reported during the specified time frame included fever (17.2%), vomiting (7.2%), seizures (10.3%), rash (2.6%), and jaundice (4.6%), while 58.2% of the participants reported no other diseases. The total number of responses for these additional health issues was 290.

**Table 3:** Frequency of infectious diseases among all types of breastfeeding practices.

Infectious diseases	Frequency (n)	Percentage (%)
Cold	185	47.4%
Pneumonia	209	53.6%
Diarrhea	237	60.8%
Ear infection	47	12.1%
Did your child experience any other disease in the past specific time frame?		
Fever	67	17.2%
Vomiting	28	7.2%
Seizures	40	10.3%
Rashes	10	2.6%
Jaundice	18	4.6%
No other disease	227	58.2%
Total	290	100%

Association of breastfeeding practices with infectious diseases outcome

Exclusive Breastfeeding

**Figure 3:**
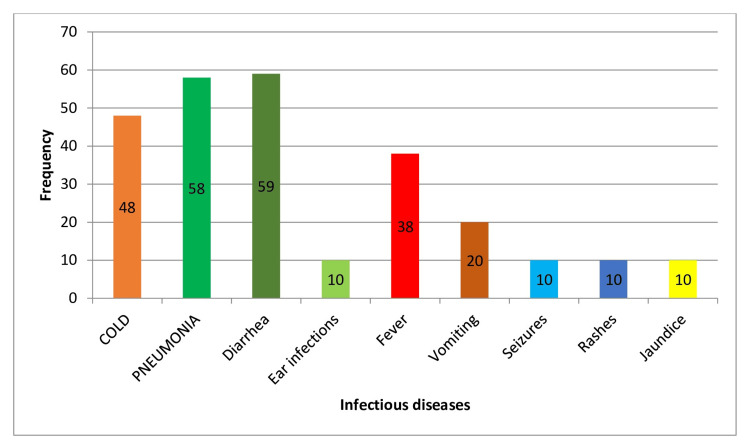
Impact of exclusive breastfeeding on infectious disease outcomes.

The data indicate that children who were exclusively breastfed had a significantly lower incidence of most infectious diseases compared to other feeding types. Specifically, the incidence rates were 32% for colds, 39.7% for pneumonia, 40% for diarrhea, and 6.8% for ear infections. The *P*-values for colds, pneumonia, diarrhea, fever, vomiting, seizures, rashes, and jaundice were all highly significant (*P* = 0.001), supporting that EBF reduces the risk of these infectious diseases. However, the *P*-value for ear infections was not significant (*P* = 0.109), indicating no significant difference in the incidence of ear infections for exclusively breastfed children compared to other groups (Table 4; Figure 3).

**Table 4:** Association of breastfeeding practices with infectious diseases outcome.

Feeding type	Infectious diseases outcome	Frequency (*n*)	Percentage (%)	*P*-value
Exclusive breastfeeding				
	Cold	48	32%	0.001
	Pneumonia	58	39.7%	0.001
	Diarrhea	59	40%	0.001
	Ear infections	10	6.8%	0.109
	Fever	38	26%	0.001
	Vomiting	20	13.6%	0.001
	Seizures	10	6.8%	0.001
	Rashes	10	6.8%	0.001
	Jaundice	10	6.8%	0.001
Partial breastfeeding				
	Cold	39	40.2%	0.10
	Pneumonia	56	57.7%	0,345
	Diarrhea	74	76.2%	0.001
	Ear infections	21	21.6%	0.001
	Fever	26	26.8%	0.001
	Vomiting	18	18.5%	0.001
	Seizures	14	14.43%	0.001
	Rashes	17	17.52%	0.001
	Jaundice	10	10.3%`	0.001
Formula feeding only				
	Cold	82	67.2%	0.001
	Pneumonia	87	71.4%	0.001
	Diarrhea	94	77.04%	0.001
	Ear infections	6	3.2%	0.04
	Fever	10	8.1%	0.001
	Vomiting	36	29.5%	0.001
	Seizures	20	16.3%	0.001
	Rashes	7	5.7%	0.001
	Jaundice	10	8%	0.001

Partial Breastfeeding

**Figure 4:**
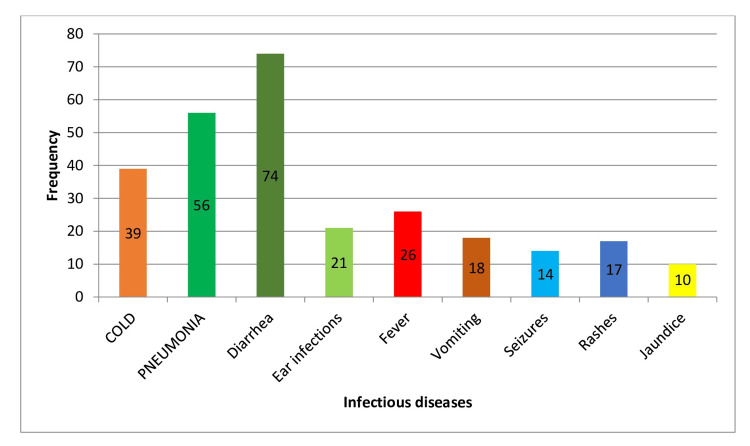
Impact of partial breastfeeding on infectious disease outcomes.

For children who were partially breastfed, the incidence rates were 40.2% for colds, 57.7% for pneumonia, 76.2% for diarrhea, and 21.6% for ear infections. The *P*-values were significant for diarrhea, ear infections, fever, vomiting, seizures, rashes, and jaundice (all *P* = 0.001), indicating a notable association between partial breastfeeding and these infectious diseases. However, the *P*-values for colds (*P* = 0.10) and pneumonia (*P* = 0.345) were not significant, suggesting no significant difference in the incidence of these diseases compared to other feeding types (Table 4; Figure 4).

Formula Feeding Only

**Figure 5:**
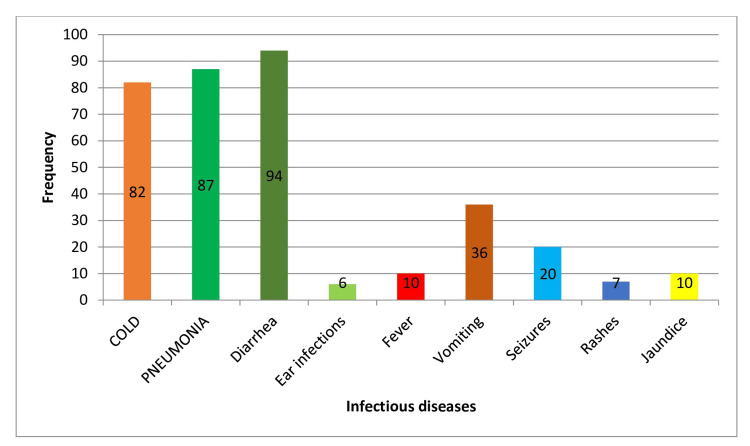
Impact of formula feeding only on infectious disease outcomes.

Children who were exclusively formula-fed had higher incidence rates of most infectious diseases, with 67.2% for colds, 71.4% for pneumonia, 77.04% for diarrhea, and 3.2% for ear infections. The *P*-values for all conditions, except ear infections (*P* = 0.04), were highly significant (*P* = 0.001), indicating a strong association between formula feeding and a higher risk of infectious diseases. This suggests that formula-fed children are more susceptible to infectious diseases compared to those who are breastfed, either exclusively or partially (Table 4; Figure 5).

Association of breastfeeding practices with cognitive development outcomes

Exclusive Breastfeeding

The data indicate that children who were exclusively breastfed showed a notably lower incidence of cognitive development challenges. Specifically, among exclusively breastfed children, only 6.1% were rated as below average in cognitive development, while 59.5% were average, and 34.2% were above average. The *P*-values for all categories were highly significant (*P* = 0.001), indicating a strong association between EBF and better cognitive development outcomes.

Partial Breastfeeding

Children who were partially breastfed also demonstrated favorable cognitive development outcomes. Among them, 28.8% were below average, and 71.1% were rated as average. Notably, there were no cases where cognitive development was rated as above average in this group. The *P*-values for below-average and average cognitive development were highly significant (*P* = 0.001). However, due to the absence of cases with above-average cognitive development, the association with partial breastfeeding in this category could not be determined.

Formula Feeding

**Figure 6:**
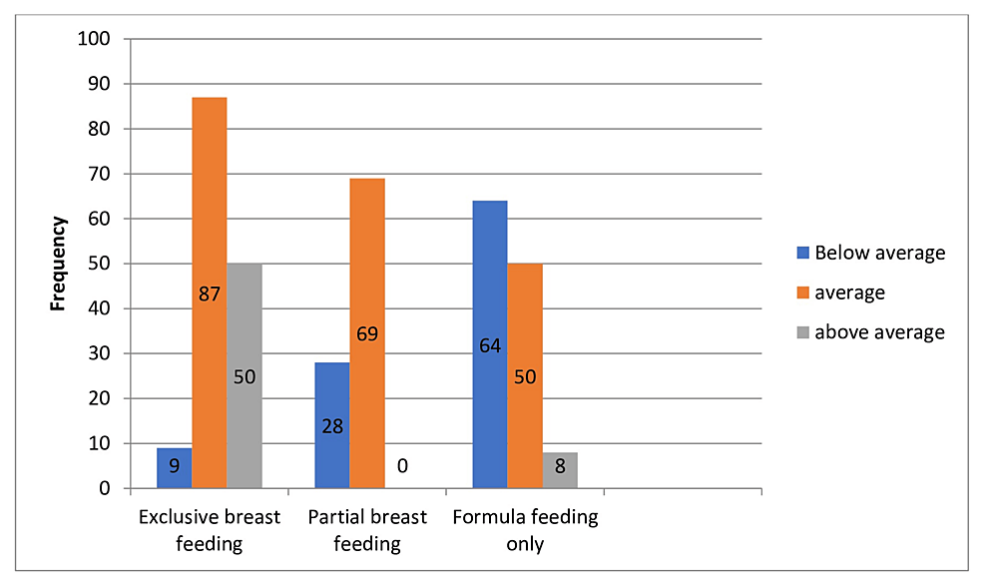
Influence of breastfeeding practices on early childhood cognitive development.

Children who were exclusively formula-fed had less favorable cognitive development outcomes compared to breastfed children. In this group, 52.4% were below average, 40.9% were average, and 6.5% were above average in cognitive development. The *P*-values for all categories were highly significant (*P* = 0.001), indicating a strong association between formula feeding and lower cognitive development outcomes(Table 5; Figure 6).

**Table 5:** Association of breastfeeding practices with cognitive development outcomes in early childhood.

Feeding type	Cognitive development outcomes in early childhood	Frequency (*n*)	Percentage (%)	*P*-value
Exclusive breastfeeding				
	Below average	9	6.1%	0.001
	Average	87	59.5%	0.001
	Above average	50	34.2%	0.001
Partial breastfeeding				
	Below average	28	28.8%	0.001
	Average	69	71.1%	0.001
	Above average	0	0	0.001
Formula feeding				
	Below average	64	52.4%	0.001
	Average	50	40.9%	0.001
	Above average	8	6.5%	0.001

Association of breastfeeding practices with developmental delay outcomes

Exclusive Breastfeeding

The data reveal that children who were exclusively breastfed exhibited a notably lower prevalence of developmental delay. Among exclusively breastfed children, 14.1% experienced developmental delay, while the majority (85.9%) did not. The *P*-value for this association was highly significant (*P* = 0.001), supporting the hypothesis that children with EBF have a lower prevalence of developmental delay.

Partial Breastfeeding

Children who were partially breastfed showed a higher prevalence of developmental delay compared to those who were exclusively breastfed. Among them, 62.8% experienced developmental delay, while 37.1% did not. The *P*-values for both groups were significant (*P* = 0.016), suggesting an association between partial breastfeeding and developmental delay, although weaker than that observed with formula feeding.

Formula Feeding

**Figure 7:**
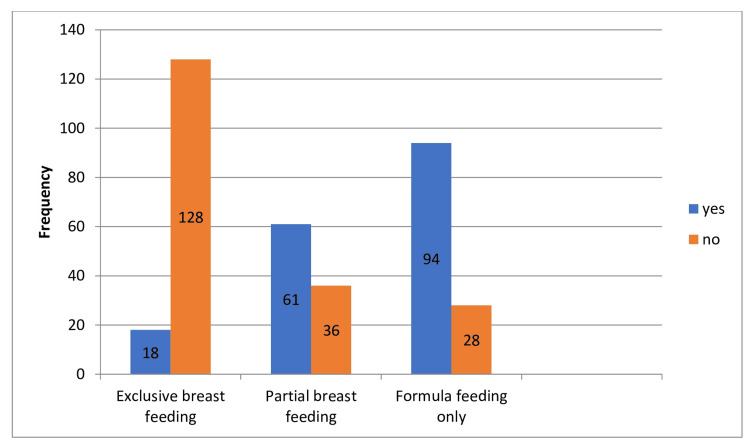
Impact of breastfeeding practices on developmental delay outcomes.

Children who were exclusively formula-fed had the highest prevalence of developmental delay among the groups. In this category, 77% of children experienced developmental delay, while 22.9% did not. The *P*-values for both groups were highly significant (*P* = 0.001), indicating a strong association between formula feeding and developmental delay (Table 6; Figure 7).

**Table 6:** Association of breastfeeding practices with developmental delay outcomes.

Feeding type	Developmental delay outcomes	Frequency (*n*)	Percentage (%)	*P*-value
Exclusive breastfeeding				
	Yes	18	14.1%	0.001
	No	128		0.001
Partial breastfeeding				
	Yes	61	62.8%	0.016
	No	36	37.1%	0.016
Formula breastfeeding				
	Yes	94	77.0%	0.001
	no	28	22.9%	0.001

## Discussion

This study explored the impact of breastfeeding practices on various health and developmental outcomes in infants and young children at Khyber Teaching Hospital, Peshawar. The findings reveal significant associations between breastfeeding practices and the incidence of infectious diseases, cognitive development outcomes, and developmental delays.

A register-based study in Uppsala County, Sweden was conducted, and it found that compared to EBF for six months or more, the absence of breastfeeding was strongly associated with an increased risk of enteric infections (adjusted incidence rate ratio [aIRR] 3.32, 95% confidence interval [CI] 2.14-5.14). Similarly, during later childhood, the absence of breastfeeding was linked to an increased risk of respiratory infections (aIRR 2.53, 95% CI 1.51-4.24). The risk of hospitalizations for infectious diseases was similar in children exclusively breastfed for four to five months and those exclusively breastfed for six months or more [19]. Our study found that children who were exclusively breastfed experienced fewer cases of colds, pneumonia, and diarrhea compared to those who were either partially breastfed or fed with formula. Specifically, the incidence rates for EBF were 32% for colds, 39.7% for pneumonia, and 40% for diarrhea. These findings are supported by significant *P*-values (*P* = 0.001), indicating a robust association between EBF and a reduced risk of these infectious diseases. This evidence supports the hypothesis that EBF provides substantial protection against infectious diseases, aligning with the WHO's recommendation for EBF during the first six months of life.

Another study examined the effects of early breastfeeding interruption on the incidence of diarrhea in infants. The findings highlight the increased risk associated with discontinuing EBF before certain milestones. Specifically, stopping EBF before three months was significantly linked to an increased likelihood of having diarrhea at six months (OR = 1.80, *P* ≤ 0.01) and between six and 12 months (OR = 1.45, *P* ≤ 0.01). Moreover, stopping breastfeeding before six months significantly raised the odds of diarrhea at six months (OR = 3.19, *P* ≤ 0.01). Additionally, formula feeding for three months or more was linked to higher odds of diarrhea between six and 12 months [20]. In our study, the prevalence of diarrhea in children who were exclusively breastfed is 40%, which is far less than in those who were partially or formula-fed. This study strongly supports our results that EBF has a lower risk of infectious diseases.

Another related study found that upper respiratory tract infections, occurring in almost one out of four infants, were identified as the most common type of infectious disease in related studies. Notably, the incidence of infectious diseases was significantly lower in the EBF group compared to the formula-feeding group (*P* = 0.005). These findings highlight the protective effects of EBF against common infections during infancy [14]. In our study, we found similar protective effects of EBF against various infectious diseases. Exclusively breastfed children exhibited significantly lower incidences of colds, pneumonia, and diarrhea compared to those who were partially breastfed or formula-fed. Specifically, the incidence rates for EBF were 32% for colds, 39.7% for pneumonia, and 40% for diarrhea. The *P*-values (*P* = 0.001) for these associations were highly significant, indicating a strong link between EBF and a reduced risk of these infectious diseases.

Recent research findings support the conclusions of the largest randomized trial in human lactation, indicating that extended EBF enhances cognitive development. This is evidenced by higher Intelligence Quotient (IQ) scores and superior academic ratings from teachers at the age of 6.5 years [21]. This supports our study, which also found that exclusively breastfed children had notably better cognitive development outcomes compared to those who were partially breastfed or formula-fed. Specifically, 34.2% of exclusively breastfed children were rated above average in cognitive development, compared to only 6.5% of formula-fed children. This significant association underscores the positive impact of EBF on early cognitive development.

A recent study has been conducted which results are consistent with the extensive randomized trial on human lactation, which identified numerous short-term and long-term benefits of EBF for children. These benefits include healthier eating habits, shorter hospital stays, favorable weight gain, lower body mass index, reduced adiposity, lower total cholesterol levels, and improved cognitive and behavioral development. Additionally, EBF contributes to the stability of metabolic levels in children with metabolic disorders [22]. This study also supports our results in which EBF is associated with better cognitive development, while formula feeding only has a higher prevalence of below-average cognitive outcomes.

A study conducted in Poland presents the first longitudinal analysis of cognitive development, aiming to assess the relationship between EBF during infancy and long-term cognitive benefits in term children. The Generalized Estimating Equation (GEE) longitudinal model indicated that EBF for six months or more was linked to an average increase of 3.8 IQ points over a seven-year follow-up period compared to mixed feeding practices. These findings confirm that EBF enhances cognitive ability in children, with beneficial effects observed even after shorter periods of breastfeeding. Overall, extended breastfeeding durations were associated with gradual improvements in cognitive development scores [23]. It is similar to our study finding which also finds that children who are exclusively breastfed have better cognitive ability compared to those who are partially and formula-fed, in whom cognitive ability is not as pronounced.

The consistent cognitive gains observed in subsequent checkups may indicate a significant biological impact of EBF on the neonatal brain. A recent study supports this notion, revealing that the development of brain electrical activity during infancy differs between breastfed infants and those fed cow’s milk or soy milk. The authors suggest that these differences in brain electrical activity could be influenced by omega-3 polyunsaturated fatty acids, which are naturally present in breast milk, along with other essential bioactive components for development [24]. In summary, our findings align with numerous related studies that demonstrate the cognitive benefits of EBF. Children who are exclusively breastfed consistently show better cognitive outcomes compared to those who are partially breastfed or fed with formula. This body of evidence underscores the importance of EBF in promoting optimal brain development and cognitive function in infants.

A nationwide cohort study was conducted in Taiwan. This study aimed to investigate the relationship between the duration of breastfeeding and four developmental domains: gross motor, fine motor, language, and personal/social skills. The study included 14,621 infants from birth to 18 months, drawn from the Taiwan Birth Cohort Study. Four developmental screening items adapted from the Denver Developmental Screening Test were found to be most suitable for children aged 15 to 18 months. The findings showed that the proportion of children achieving specific milestones increased consistently with longer durations of breastfeeding. The adjusted odds ratios for the risk of developmental delay, comparing the longest duration of breastfeeding to never being breastfed, were 0.69 (95% CI 0.57-0.83) for gross motor, 0.64 (95% CI 0.53-0.77) for fine motor, 0.74 (95% CI 0.60-0.91) for language, and 0.76 (95% CI 0.64-0.90) for personal/social skills. The inverse association between breastfeeding duration and developmental delay was significant regardless of when mothers returned to work. Protection against developmental delays was notably significant for children breastfed for more than six months. These findings support the hypothesis that longer breastfeeding duration is positively related to neurodevelopment in young children [25].

Our study's findings align with previous research on the relationship between breastfeeding duration and developmental outcomes. For instance, the Taiwan Birth Cohort Study indicated that longer breastfeeding durations are associated with reduced risks of developmental delays in various domains, such as gross motor, fine motor, language, and personal/social skills. Similarly, our data demonstrates that EBF is linked to a significantly lower prevalence of developmental delays. Specifically, only 14.1% of exclusively breastfed children experienced developmental delays compared to 62.8% of partially breastfed and 77% of formula-fed children. The *P*-values were highly significant for EBF (*P* = 0.001) and formula feeding (*P* = 0.001), underscoring the robust association between EBF and better developmental outcomes. Partial breastfeeding also showed a significant association with developmental delays (*P* = 0.016), though the effect was less pronounced than in formula-fed children.

Limitations

Despite the significant findings, this study has several limitations that should be acknowledged. First, the research was conducted in a single city and at one hospital (Khyber Teaching Hospital, Peshawar), which may limit the generalizability of the results to other populations and settings. The sample size of 390, while adequate for a cross-sectional study, might not capture the full diversity of breastfeeding practices and their outcomes across different socioeconomic and cultural backgrounds. Additionally, the cross-sectional design of the study restricts our ability to establish causal relationships between breastfeeding practices and the observed health and developmental outcomes. Longitudinal studies would be needed to confirm these associations over time. Conducting longitudinal research would allow for the observation of breastfeeding practices and their outcomes across different stages of child development, providing stronger evidence for causal inferences

Recommendations for future research

Future studies should consider longitudinal designs to track the long-term impact of breastfeeding practices on health and developmental outcomes beyond early childhood. Additionally, exploring the underlying mechanisms through which breastfeeding influences cognitive and motor development, as well as its protective effects against infectious diseases, could provide deeper insights and strengthen the evidence base for breastfeeding promotion initiatives.

## Conclusions

This study explored the impact of breastfeeding practices on various health and developmental outcomes in infants and young children at Khyber Teaching Hospital, Peshawar. The findings reveal significant associations between breastfeeding practices and the incidence of infectious diseases, cognitive development outcomes, and developmental delays. EBF was associated with a notably lower incidence of infectious diseases such as colds, pneumonia, diarrhea, and fever compared to partial and formula feeding. Specifically, EBF showed significant protective effects against these diseases, highlighting its importance for infant health. Regarding cognitive development, children who were exclusively breastfed demonstrated higher percentages of above-average cognitive outcomes, while formula-fed children had a higher prevalence of below-average cognitive outcomes. This suggests that EBF positively influences cognitive development in early childhood. Furthermore, the study found that EBF was linked to the lowest prevalence of developmental delays, while formula feeding was associated with the highest prevalence. These results underscore the critical role of EBF in promoting optimal health and developmental outcomes in infants and young children, supporting existing recommendations for EBF during the first six months of life. Overall, this research contributes valuable insights into the multifaceted benefits of breastfeeding, reinforcing its significance for child health and development.

## Appendices

Appendix

Questionnaire: Impact of Breastfeeding on Child Health Outcomes

1.Child's Age (in months):

2.Mother's Education Level:

3.Did you breastfeed your child?

4.Breastfeeding Duration (in months):

5. Exclusive breastfeeding

6. Partial breastfeeding (combination of breastfeeding and formula feeding)

7 .Formula feeding only

8.Has your child experienced any of the following infectious diseases in the past [specify time frame]?

 Respiratory infections (e.g., cold, pneumonia)

 Gastrointestinal infections (e.g., diarrhea, gastroenteritis)

 Ear infections

 Other (please specify): [______]

Cognitive Development Outcomes:

Cognitive Scale

Does the child respond to visual and auditory stimuli? (Yes/No/Sometimes)

Can the child remember the location of hidden objects? (Yes/No/Sometimes)

Does the child show interest in exploring and manipulating objects? (Yes/No/Sometimes)

Is the child able to solve simple problems through trial-and-error? (Yes/No/Sometimes)

Does the child understand that objects continue to exist even when out of sight? (Yes/No/Sometimes)

Above Average: 8-10 points

Average: 4-7 points

Below Average: 0-3 points

9.How would you rate your child's cognitive development compared to other children of the same age?

 Below average

 Average

 above average

 12. How would you rate your child’s gross motor skill development compared to other children of the same age? (e.g., crawling, walking, running)

- Below average

- Average

- Above average

13.How would you rate your child’s fine motor skill development compared to other children of the same age? (e.g., grasping objects, drawing)

Below average

Average

Above average

14.At what age did your child first achieve the following gross motor milestones? (Please specify in months)

Rolling over: [Specify months]

Sitting up without support: [Specify months]

Crawling: [Specify months]

Standing without support: [Specify months]

Walking independently: [Specify months]

15.At what age did your child first achieve the following fine motor milestones? (Please specify in months)

Reaching and grasping objects: [Specify months]

Transferring objects from one hand to another: [Specify months]

Pincer grasp (thumb and forefinger): [Specify months]

Stacking blocks or similar activities: [Specify months]
